# Synthesis and structure of benzyl 4-{[4-(hex­yloxy)benzo­yl]­oxy}benzoate

**DOI:** 10.1107/S2056989026008728

**Published:** 2026-08-28

**Authors:** P. Roopadevi, H. T. Srinivasa, H. C. Devarajegowda, R. Karthik, B. S. Palakshamurthy

**Affiliations:** a Department of Physics Yuvaraja's College University of Mysore,Mysore-570005 Karnataka India; b Raman Research Institute, C V Raman Avenue Sadashivanagar Bangalore-560080 Karnataka India; c Department of Physics, Vidyavardhaka College of Engineering, Mysore, 570002, Karnataka, India; d Department of PG Studies and Research in Physics Albert Einstein Block UCS Tumkur University, Tumkur Karnataka-572103 India; University of Aberdeen, United Kingdom

**Keywords:** crystal structure, Hirshfeld surface, 4-hex­yloxy, 4-benzoyl­oxybenzolate

## Abstract

In the title compound, C_27_H_28_O_5_, the aromatic ring of the 4-hexyl­oxybenzoyl moiety and the central benzoate ring are twisted relative to one another, subtending a dihedral angle of 39.7 (2)°. The pendant benzyl aromatic ring is nearly orthogonal to the central benzoate ring, forming a dihedral angle of 89.3 (2)°. The hex­yloxy chain adopts an extended, approximately all-*anti* conformation and two short intra­molecular C—H⋯O contacts are observed. In the extended structure, C—H⋯π inter­actions help to consolidate the packing. Hirshfeld surface analysis and the corresponding two-dimensional fingerprint plots indicate that H⋯H contacts constitute the largest contribution to the crystal surface, accounting for 55.4%, followed by C⋯H/H⋯C contacts (22.9%) and O⋯H/H⋯O contacts (16.3%).

## Abstract 2

In the title compound, the aromatic ring of the 4-hexyl­oxybenzoyl moiety and the central benzoate ring are twisted relative to one another, subtending a dihedral angle of 39.7 (2)°. The hex­yloxy chain adopts an extended, approximately all-*anti* conformation and two short intra­molecular C—H⋯O contacts are observed. In the extended structure, C—H⋯π inter­actions help to consolidate the packing.

## 1. Chemical context

Derivatives of 4-hy­droxy­benzoic acid have been associated with anti­microbial, anti­oxidant and anti-inflammatory properties, while naturally occurring alk­oxy-substituted benzoic acid derivatives can inhibit inflammatory responses (Manuja *et al.*, 2013 ▸; Chen *et al.*, 2008 ▸). Esterification can modify lipophilicity, membrane transport and susceptibility to enzymatic hydrolysis; accordingly, several alkyl and aryl benzoates have been investigated as anti­mycobacterial prodrugs (Pais *et al.*, 2022 ▸). Benzyl and other aromatic ester derivatives have also been examined for anti­microbial and anti­oxidant activities, demonstrating that the nature of the aromatic substituent and ester group can influence biological behavior (Matin *et al.*, 2016 ▸; Kumar *et al.*, 2015 ▸). In materials chemistry, phenyl-benzoate systems containing multiple aromatic rings, ester linkages and flexible terminal alk­oxy chains represent an established design for calamitic liquid crystals. Studies of related homologous series show that the length of the terminal alk­oxy chain, mol­ecular rigidity and terminal substituents strongly affect melting behaviour and the formation and stability of nematic or smectic phases (Patel & Chauhan, 2016 ▸; Jagtap & Chauhan, 2016 ▸; Balkanli *et al.*, 2021 ▸; Alamro *et al.*, 2024 ▸). Aromatic esters are additionally of inter­est because their mol­ecular symmetry, rotational freedom and π-stacking tendencies, which influence crystallization and thermal properties (Ravotti *et al.*, 2020 ▸). As part of our studies in this area, we now describe the synthesis and structure of the title compound, C_27_H_25_O_5_ (**I**).
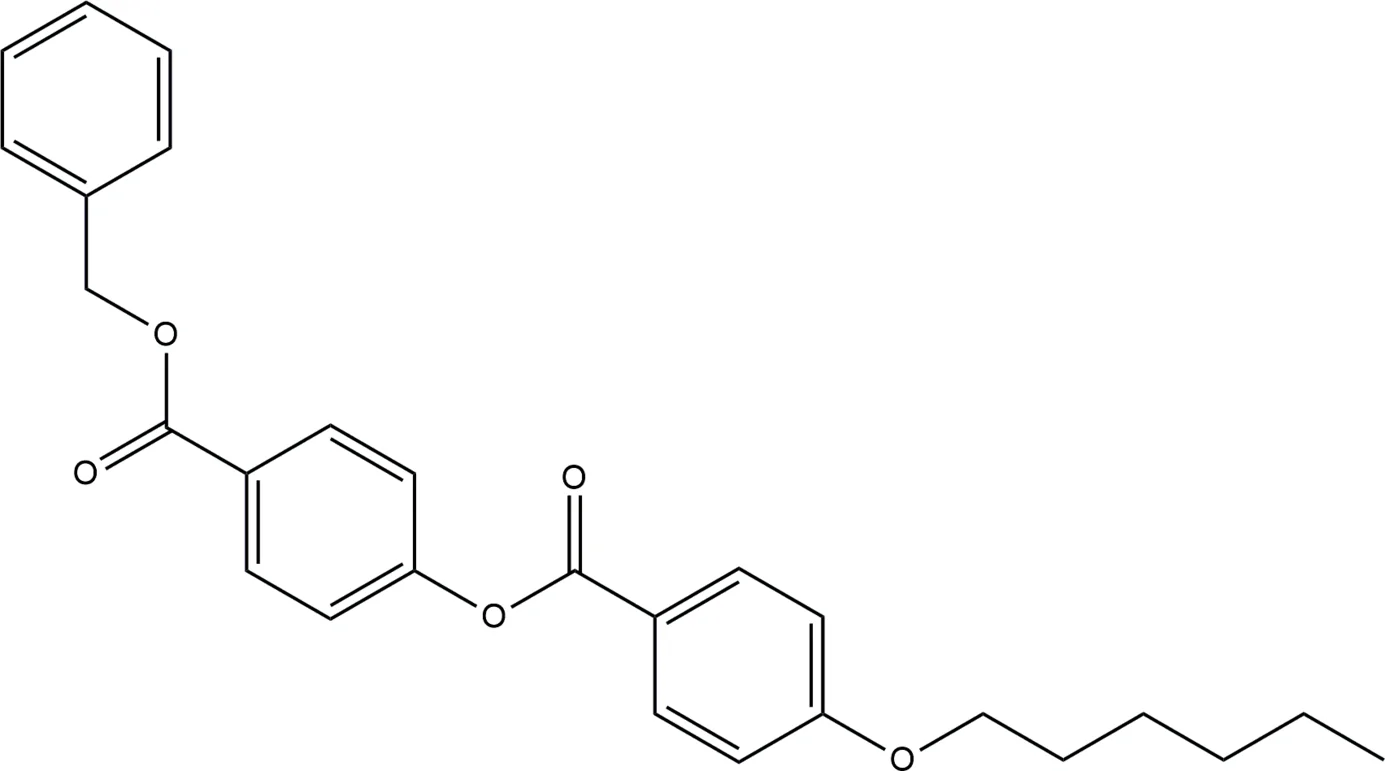


## 2. Structural commentary

**Figure 1:**
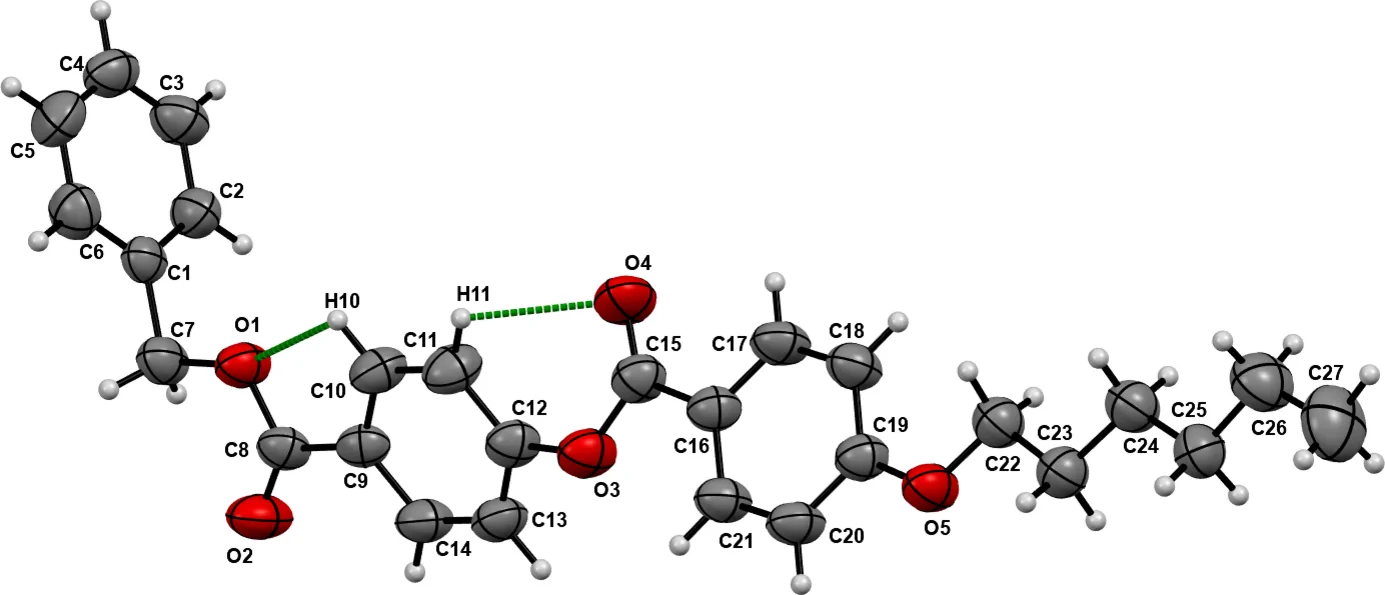
The mol­ecular structure of (**I**) drawn with 50% probability ellipsoids with intra­molecular hydrogen bonds and short contacts shown as green and orange dashed lines.

Compound (**I**) crystallizes in space group *P*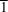, with one mol­ecule in the asymmetric unit (Fig. 1 ▸). The conformation is governed principally by the relative orientations of the three aromatic rings and the inter­vening ester linkages. The benzyl phenyl ring (C1–C6) is almost perpendicular to the central benzoate ring (C9–C14), forming a dihedral angle of 89.26 (2)°. The 4-hexyl­oxybenzoyl ring (C16–C21) is inclined to the central ring by 39.70 (12)°, while it forms a dihedral angle of 74.50 (5)° with the benzyl phenyl ring. The mol­ecule therefore adopts a markedly non-coplanar overall conformation. The C—C bond associated with the benzyl ester linkage adopts a near-*anti* arrangement, as indicated by the C1—C7—O1—C8 torsion angle of −164.86 (14)°. The ester linkage connecting the central and 4-hexyl­oxybenzoyl fragments is more extended, with a C12—O3—C15—C16 torsion angle of −178.07 (14)°. The carbonyl plane associated with C15 is inclined by 7.85 (13)° to the 4-hexyl­oxybenzoyl ring but by 33.20 (12)° to the central aromatic ring, showing that conjugation is maintained preferentially on the benzoyl side of the ester group. The hex­yloxy substituent also adopts an extended orientation relative to its attached aromatic ring, with a C19—O5—C22—C23 torsion angle of 177.28 (15)°. The subsequent alkyl-chain torsion angles C22—C23—C24—C25 and C23—C24—C25—C26 are 179.98 (17) and −174.58 (18)°, respectively, confirming an approximately all-*anti* chain conformation. C10—H10⋯O1 and C11—H11⋯O4 intra­molecular short contacts (Table 1 ▸) may help to consolidate the mol­ecular conformation.

## 3. Supra­molecular features

**Figure 2:**
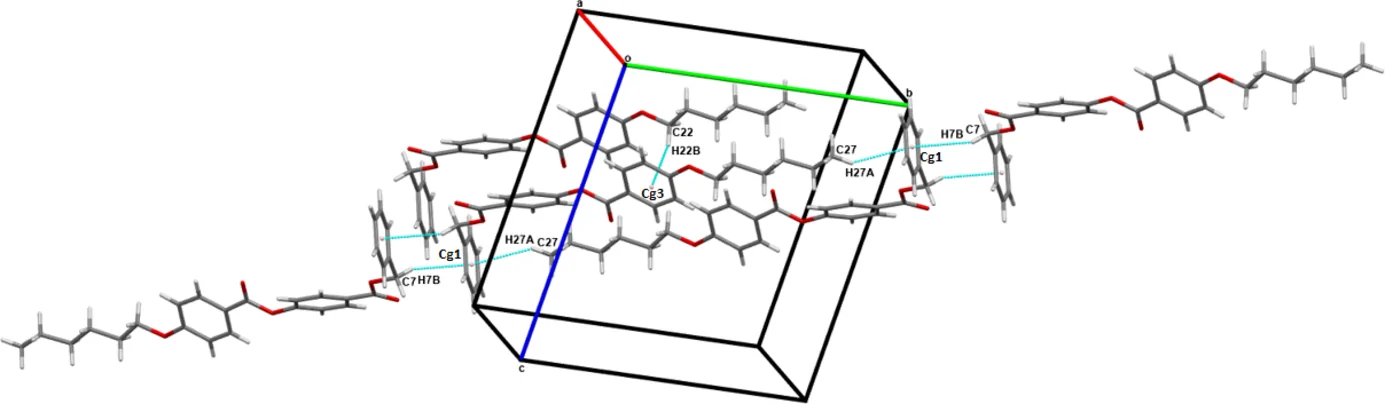
The packing of (I) with C—H⋯π inter­actions shown as blue dotted lines.

In the extended structure of (**I**), the packing is reinforced by three C—H⋯π contacts involving C7—H7*B*⋯*Cg*1, C22—H22*B*⋯*Cg*3 and C27—H27*A*⋯*Cg*1, where *Cg*1 and *Cg*3 represent the centroids of the C1–C6 and C16–C21 aromatic rings, respectively (Fig. 2 ▸ and Table 1 ▸).

## 4. Hirshfeld surface analysis

**Figure 3:**
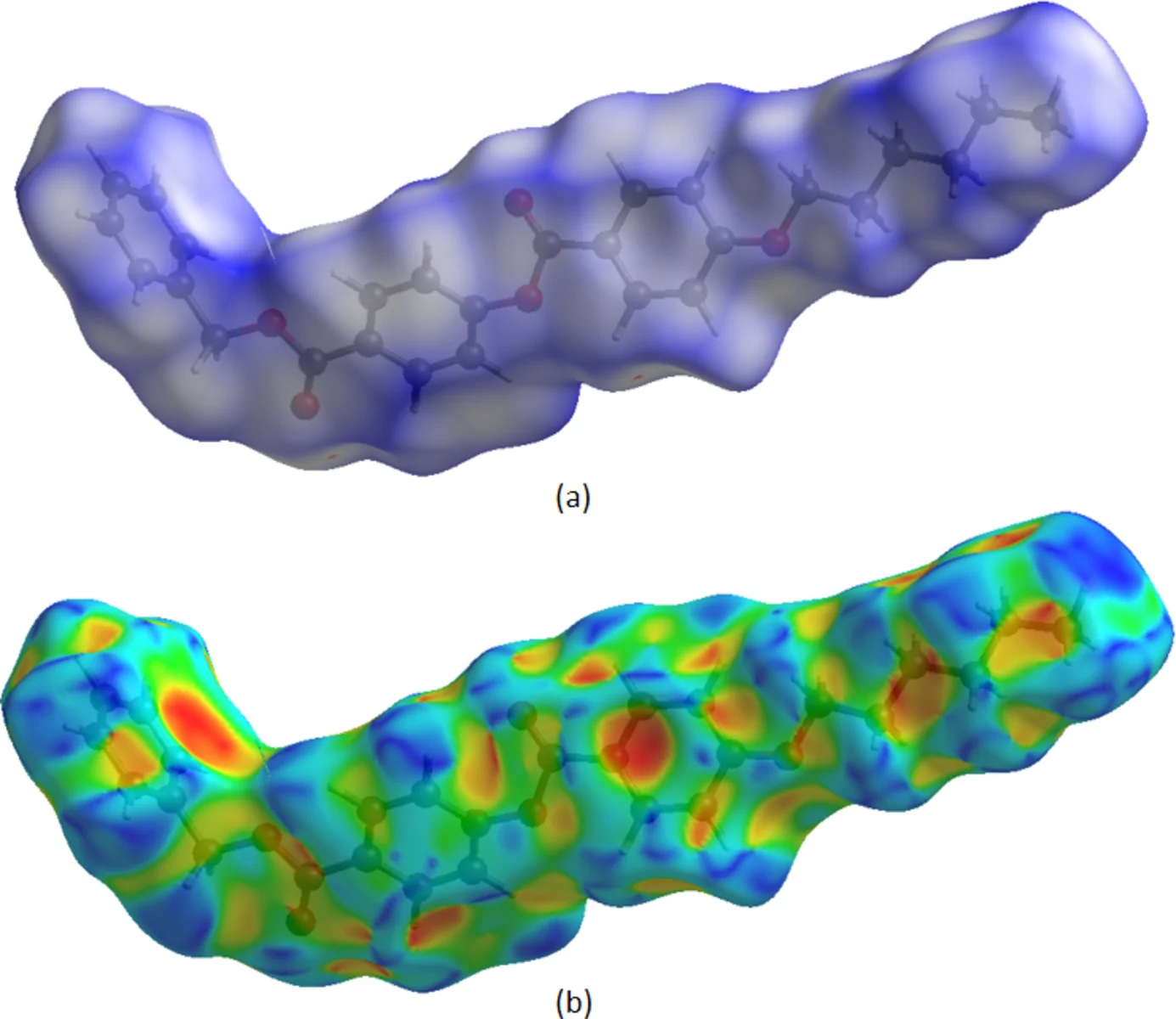
The Hirshfeld surface view of the title mol­ecule plotted over (*a*) *d*_norm_ and (*b*) shape index.

**Figure 4:**
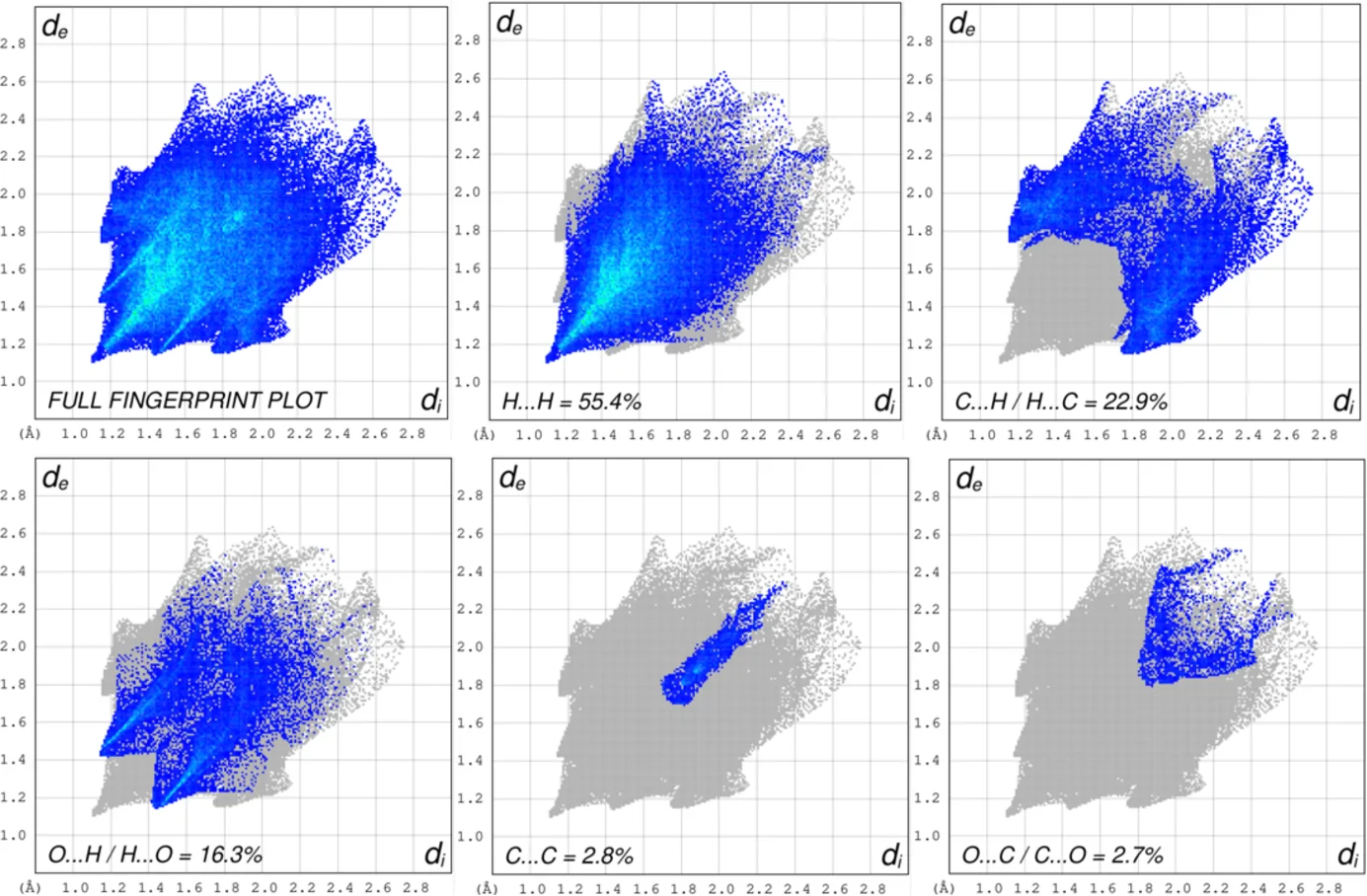
The two-dimensional fingerprint plots for (**I**) showing H⋯H (55.4%), C⋯H/H⋯C (22.9%), O⋯H/H⋯O (16.3%), C⋯C(2.8%), and O⋯C/C⋯O(2.7%) contacts.

A Hirshfeld surface analysis was carried out for (**I**) using *Crystal Explorer* 17.5 (Spackman *et al.*, 2021 ▸) to further qu­antify the inter­molecular inter­actions listed in Table 1 ▸. The three-dimensional Hirshfeld surfaces plotted over *d*_norm_ and shape-index are shown in Fig. 3 ▸. The red spots around the aromatic rings of the mol­ecule signifies the presence of centroids in Fig. (3*b*). The two-dimensional fingerprint plots (Fig. 4 ▸) indicate that the most prominent contributions for the Hirshfeld surfaces are from H⋯H (55.4%), C⋯H/H⋯C (22.9%), O⋯H/H⋯O (16.3%) contacts with C⋯C (2.8%), and O⋯C/C⋯O (2.7%) being less significant.

**Figure 5:**
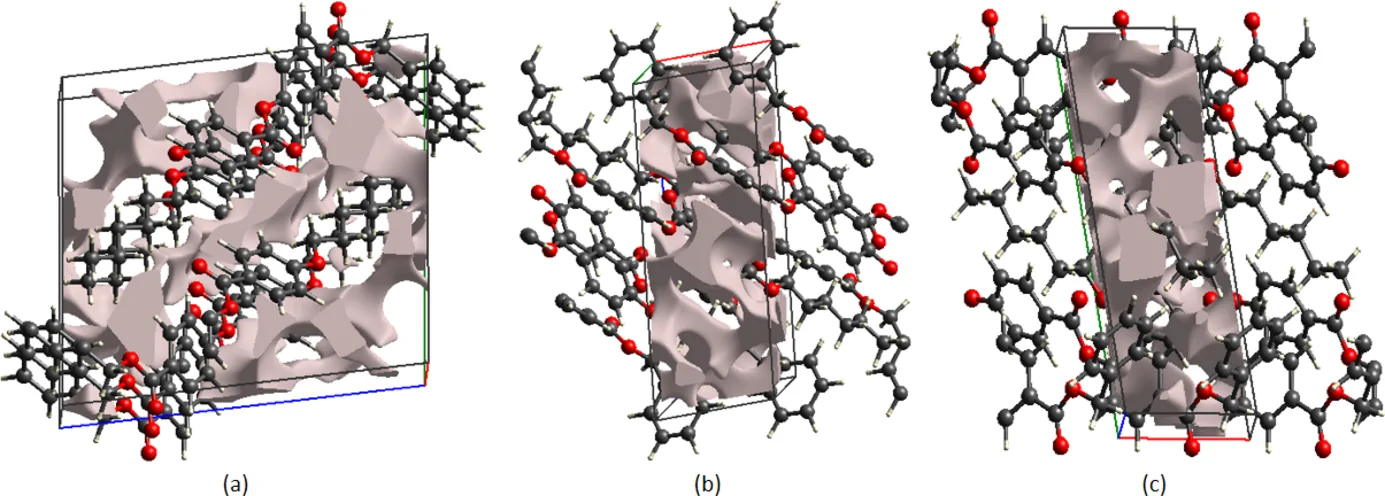
The crystal voids in (**I**) viewed along the *a*, *b* and *c* axes.

**Figure 6:**
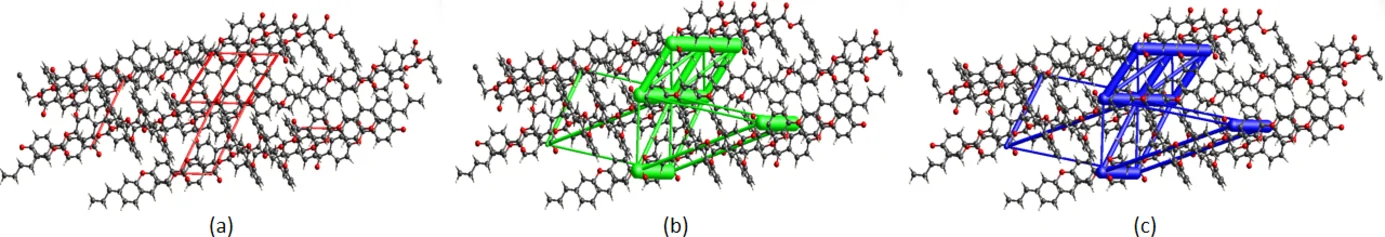
The energy framework topology of (**I**) generated for (*a*) Coulomb inter­action energy, (*b*) dispersion inter­action energy and (*c*) total inter­action energy.

A crystal void analysis was performed using a 0.002 a.u. electron-density isosurface. The calculated void volume (Fig. 5 ▸) and surface area are 143.5 Å^3^ and 482.5 Å^2^, respectively. Relative to the unit-cell volume of 1169.3 (7) Å^3^, the voids occupy approximately 12.3% of the unit cell, indicating a modest amount of unoccupied space within the crystal structure. The relatively high surface-area-to-volume ratio suggests that the void regions have an irregular and extended boundary rather than a compact spherical shape. Inter­action energies (Fig. 6 ▸) for (**I**) using the basis set B3LYP\631-G(d,p) for mol­ecular pairs within the cluster of 3.8 Å radius, gave *E*_ele_ = −49.7, *E*_pol_ = −19.5, *E*_dis_ = −293.4 and *E*_rep_ = 117 kJ mol^−1^.

## 5. Database survey

A search of the Cambridge Structural Database (CSD version 6.01, March 2026; Groom *et al.*, 2016 ▸) for structures containing the 4-hexyl­oxybenzoate fragment returned six entries, of which three were selected as close matches: CSD refcodes GIKBOT01 (Kuz’mina *et al.*, 2013 ▸), JITNAB (Haase *et al.*, 1991 ▸) and ZIFLOP (Iki & Hori, 1995 ▸). A broader search based on related benzoate and aromatic-ester fragments identified MESQAG (Brown *et al.*, 2021 ▸) and UBAJOZ (Tabuchi *et al.*, 2016 ▸). The dihedral angles between the ester-linked aromatic rings in GIKBOT01, JITNAB and ZIFLOP are 56.4, 66.2 and 1.0°, respectively, showing substantial variation from nearly coplanar to strongly twisted conformations. In contrast, the carbonyl group remains close to the plane of the 4-hexyl­oxybenzoate ring, with corresponding inter­planar angles of 5.6, 7.4 and 0.9°. The C(ar)—O—C—C torsion angles of 166.0, 176.4 and 179.2° indicate predominantly extended hex­yloxy chains. In MESQAG, the ester-linked aromatic rings subtend a dihedral angle of 25.4°, while UBAJOZ, a lower pent­yloxy homologue, has a carboxyl-group inclination of 2.0° and a C(ar)—O—C—C torsion angle of 168.6°. In (**I**), the 4-hexyl­oxybenzoate and central benzoate rings form a dihedral angle of 39.7°, within the range observed for the closest database structures. Thus, the title mol­ecule combines an inter­mediate twist across the diaryl ester linkage with an almost orthogonal benzyl group and an extended terminal alk­oxy chain.

## 6. Synthesis and crystallization

A mixture of 4-(hex­yloxy)benzoic acid (0.223 g, 1 eq), benzyl 4-hy­droxy­benzoate (0.228 g, 1.0 eq) in dry di­chloro­methane with di­cyclo­hexyl­carbodi­imide (DCC) (0.210 g, 1.2 eq) and a catalytic amount of di­methyl­amino­pyrimidine (DMAP) were stirred at room temperature for 6 hrs. The side product *N*,*N*-di­cyclo­hexyl urea formed was filtered off and the filtrate diluted with di­chloro­methane (25ml). This solution was washed successively with 5% aqueous acetic acid solution (2 × 25ml) and water (2 × 25ml) and dried over sodium sulfate. The residue obtained after solvent removal was chromatographed on silica gel using chloro­form as eluent. Removal of solvent from the eluate afforded a white material, which was recrystallized from chloro­form solution. Yield: 0.145g (75%); elemental analysis calculated: C, 74.98; H, 6.53; O, 18.50% found is C, 75.02; H, 6.55%. ^1^H NMR (ppm, CDCl_3_): 8.13 (*m*, 4H, Ar-H), 7.62 (*m*, 2H, Ar-H), 7.32 (*s*, 5H, Ar-H), 7.07 (*m*, 2H, Ar-H), 5.2 (*s*, 2H, Ar-CH_2_–), 4.01 (*t*, 2H, *J* = 6.5Hz, –OCH_2_–), 1.72–1.26 (*m*, 8H, alkyl-H), 0.92 (*t*, 3H, *J* = 4.5Hz, –CH_3_).

## 7. Refinement

Crystal data, data collection and structure refinement details are summarized in Table 2 ▸. All hydrogen atoms were positioned with idealized geometry and refined using a riding model with C—H = 0.93–0.97 Å and *U*_iso_(H) = 1.2 *U*_eq_(C) or 1.5 *U*_eq_(methyl C).

## Supplementary Material

Crystal structure: contains datablock(s) I. DOI: 10.1107/S2056989026008728/hb8247sup1.cif

Structure factors: contains datablock(s) I. DOI: 10.1107/S2056989026008728/hb8247Isup2.hkl

Supporting information file. DOI: 10.1107/S2056989026008728/hb8247Isup3.cml

CCDC reference: 2582616

Additional supporting information:  crystallographic information; 3D view; checkCIF report

## Figures and Tables

**Table 1:** Hydrogen-bond geometry (Å, °) *Cg*1 and *Cg*3 represent the centroids of the C1–C6 and C16–C21 aromatic rings, respectively.

*D*—H⋯*A*	*D*—H	H⋯*A*	*D*⋯*A*	*D*—H⋯*A*
C10—H10⋯O1	0.93	2.41	2.730 (2)	100
C11—H11⋯O4	0.93	2.40	2.851 (2)	109
C7—H7*B*⋯*Cg*1^i^	0.97	2.92	3.602 (2)	128
C22—H22*B*⋯*Cg*3^ii^	0.97	2.99	3.810 (2)	143
C27—H27*A*⋯*Cg*1^iii^	0.96	2.98	3.791 (3)	143

Symmetry codes: (i) 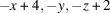; (ii) 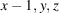; (iii) 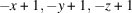.

**Table 2:** Experimental details

Crystal data	
Chemical formula	C_27_H_28_O_5_
*M*_r_	432.49
Crystal system, space group	Triclinic, *P* 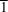
Temperature (K)	258
*a*, *b*, *c* (Å)	5.435 (2), 14.445 (5), 15.277 (5)
α, β, γ (°)	95.104 (11), 95.693 (12), 99.725 (12)
*V* (Å^3^)	1169.3 (7)
*Z*	2
Radiation type	Mo *K*α
μ (mm^−1^)	0.08
Crystal size (mm)	0.38 × 0.32 × 0.27

Data collection	
Diffractometer	Bruker SMART APEXII CCD
Absorption correction	Multi-scan (*SADABS*; Krause et al., 2015 ▸)
*T*_min_, *T*_max_	0.965, 0.975
No. of measured, independent and observed [*I* > 2σ(*I*)] reflections	30609, 6801, 4287
*R*_int_	0.041
(sin θ/λ)_max_ (Å^−1^)	0.705

Refinement	
*R*[*F*^2^ > 2σ(*F*^2^)], *wR*(*F*^2^), *S*	0.062, 0.155, 1.03
No. of reflections	6801
No. of parameters	289
H-atom treatment	H-atom parameters constrained
Δρ_max_, Δρ_min_ (e Å^−3^)	0.17, −0.19

Computer programs: *APEX2* and *SAINT* (Bruker, 2017 ▸), *SHELXT2018/3* (Sheldrick, 2015*a* ▸), *SHELXL2019/2* (Sheldrick, 2015*b* ▸), *Mercury* (Macrae *et al.*, 2020 ▸) and *publCIF* (Westrip, 2010 ▸).

## supplementary crystallographic information

### Benzyl 4-{[4-(hexyloxy)benzoyl]oxy}benzoate Crystal data

**Table:**

C_27_H_28_O_5_	*Z* = 2
*M**_r_* = 432.49	*F*(000) = 460
Triclinic, *P*1	*D*_x_ = 1.228 Mg m^−^^3^
*a* = 5.435 (2) Å	Mo *K*α radiation, λ = 0.71075 Å
*b* = 14.445 (5) Å	Cell parameters from 4287 reflections
*c* = 15.277 (5) Å	θ = 3.0–30.0°
α = 95.104 (11)°	µ = 0.08 mm^−^^1^
β = 95.693 (12)°	*T* = 258 K
γ = 99.725 (12)°	PRISM, colourless
*V* = 1169.3 (7) Å^3^	0.38 × 0.32 × 0.27 mm

### Benzyl 4-{[4-(hexyloxy)benzoyl]oxy}benzoate Data collection

**Table:**

Bruker SMART APEXII CCD diffractometer	6801 independent reflections
Radiation source: fine-focus sealed tube	4287 reflections with *I* > 2σ(*I*)
Graphite monochromator	*R*_int_ = 0.041
Detector resolution: 1.09 pixels mm^-1^	θ_max_ = 30.1°, θ_min_ = 2.9°
φ and Ω scans	*h* = −7→7
Absorption correction: multi-scan (SADABS; Krause et al., 2015)	*k* = −20→18
*T*_min_ = 0.965, *T*_max_ = 0.975	*l* = −21→21
30609 measured reflections	

### Benzyl 4-{[4-(hexyloxy)benzoyl]oxy}benzoate Refinement

**Table:**

Refinement on *F*^2^	Primary atom site location: structure-invariant direct methods
Least-squares matrix: full	Secondary atom site location: difference Fourier map
*R*[*F*^2^ > 2σ(*F*^2^)] = 0.062	Hydrogen site location: inferred from neighbouring sites
*wR*(*F*^2^) = 0.155	H-atom parameters constrained
*S* = 1.03	*w* = 1/[σ^2^(*F*_o_^2^) + (0.0557*P*)^2^ + 0.2511*P*] where *P* = (*F*_o_^2^ + 2*F*_c_^2^)/3
6801 reflections	(Δ/σ)_max_ < 0.001
289 parameters	Δρ_max_ = 0.17 e Å^−^^3^
0 restraints	Δρ_min_ = −0.19 e Å^−^^3^
0 constraints	

### Benzyl 4-{[4-(hexyloxy)benzoyl]oxy}benzoate Special details

**Table:**

Geometry. All esds (except the esd in the dihedral angle between two l.s. planes) are estimated using the full covariance matrix. The cell esds are taken into account individually in the estimation of esds in distances, angles and torsion angles; correlations between esds in cell parameters are only used when they are defined by crystal symmetry. An approximate (isotropic) treatment of cell esds is used for estimating esds involving l.s. planes.

### Benzyl 4-{[4-(hexyloxy)benzoyl]oxy}benzoate Fractional atomic coordinates and isotropic or equivalent isotropic displacement parameters (Å^2^)

**Table:**

	*x*	*y*	*z*	*U*_iso_*/*U*_eq_	
O1	1.6426 (2)	0.07235 (8)	0.81958 (7)	0.0607 (3)	
O3	0.8144 (2)	0.18094 (9)	0.54784 (8)	0.0666 (3)	
O2	1.4848 (3)	−0.07220 (9)	0.75109 (9)	0.0782 (4)	
O5	0.0280 (2)	0.33927 (9)	0.30299 (8)	0.0678 (3)	
O4	0.8199 (3)	0.31815 (10)	0.63289 (8)	0.0808 (4)	
C1	1.9292 (3)	0.10443 (11)	0.95172 (10)	0.0493 (4)	
C9	1.3131 (3)	0.05972 (11)	0.70564 (9)	0.0499 (4)	
C20	0.3129 (4)	0.24706 (12)	0.35407 (11)	0.0612 (4)	
H20	0.269039	0.208193	0.300996	0.073*	
C2	1.7985 (3)	0.11428 (12)	1.02367 (12)	0.0610 (4)	
H2	1.643292	0.075687	1.024035	0.073*	
C4	2.1198 (4)	0.23668 (13)	1.09631 (12)	0.0664 (5)	
H4	2.182960	0.281542	1.144260	0.080*	
C21	0.4898 (3)	0.22655 (11)	0.41635 (11)	0.0589 (4)	
H21	0.564236	0.173890	0.405440	0.071*	
C8	1.4860 (3)	0.01142 (11)	0.75952 (10)	0.0521 (4)	
C16	0.5581 (3)	0.28465 (11)	0.49609 (10)	0.0539 (4)	
C6	2.1602 (3)	0.16175 (14)	0.95446 (13)	0.0660 (5)	
H6	2.254073	0.156401	0.907165	0.079*	
C14	1.1074 (3)	0.00556 (12)	0.65275 (11)	0.0601 (4)	
H14	1.081061	−0.059951	0.650674	0.072*	
C3	1.8927 (4)	0.18019 (13)	1.09529 (12)	0.0667 (5)	
H3	1.800466	0.185913	1.142980	0.080*	
C7	1.8234 (3)	0.03205 (12)	0.87481 (11)	0.0604 (4)	
H7A	1.956701	0.017121	0.841247	0.073*	
H7B	1.741047	−0.025514	0.895508	0.073*	
C13	0.9418 (3)	0.04852 (12)	0.60325 (11)	0.0617 (4)	
H13	0.802861	0.012147	0.568504	0.074*	
C10	1.3506 (3)	0.15727 (12)	0.70739 (11)	0.0590 (4)	
H10	1.487719	0.193853	0.742930	0.071*	
C17	0.4428 (4)	0.36262 (12)	0.51056 (11)	0.0622 (4)	
H17	0.487715	0.401884	0.563367	0.075*	
C12	0.9833 (3)	0.14575 (12)	0.60549 (10)	0.0554 (4)	
C11	1.1885 (3)	0.20093 (12)	0.65745 (12)	0.0645 (5)	
H11	1.216235	0.266361	0.658564	0.077*	
C23	−0.2579 (3)	0.42209 (13)	0.22933 (12)	0.0635 (4)	
H23A	−0.390594	0.366954	0.221911	0.076*	
H23B	−0.161995	0.418689	0.179229	0.076*	
C19	0.1984 (3)	0.32520 (11)	0.36921 (10)	0.0555 (4)	
C25	−0.5466 (4)	0.51750 (13)	0.14900 (13)	0.0674 (5)	
H25A	−0.690556	0.466653	0.143773	0.081*	
H25B	−0.458686	0.508887	0.097466	0.081*	
C18	0.2636 (4)	0.38315 (12)	0.44854 (11)	0.0616 (4)	
H18	0.187212	0.435259	0.459687	0.074*	
C22	−0.0893 (3)	0.42082 (13)	0.31261 (12)	0.0631 (4)	
H22A	0.037523	0.477795	0.322945	0.076*	
H22B	−0.186202	0.418017	0.362624	0.076*	
C15	0.7438 (3)	0.26666 (13)	0.56663 (11)	0.0590 (4)	
C5	2.2540 (4)	0.22708 (15)	1.02661 (15)	0.0764 (5)	
H5	2.410880	0.264954	1.027520	0.092*	
C24	−0.3743 (4)	0.51068 (14)	0.23047 (13)	0.0691 (5)	
H24A	−0.467955	0.513670	0.281196	0.083*	
H24B	−0.239926	0.565277	0.238672	0.083*	
C26	−0.6385 (5)	0.61033 (15)	0.14930 (16)	0.0867 (6)	
H26A	−0.715063	0.620716	0.203206	0.104*	
H26B	−0.494380	0.660514	0.150885	0.104*	
C27	−0.8227 (5)	0.6180 (2)	0.0728 (2)	0.1078 (8)	
H27A	−0.870148	0.679130	0.078621	0.162*	
H27B	−0.747822	0.609865	0.018914	0.162*	
H27C	−0.968994	0.569979	0.071350	0.162*	

### Benzyl 4-{[4-(hexyloxy)benzoyl]oxy}benzoate Atomic displacement parameters (Å^2^)

**Table:**

	*U*^11^	*U*^22^	*U*^33^	*U*^12^	*U*^13^	*U*^23^
O1	0.0742 (8)	0.0520 (6)	0.0519 (6)	0.0168 (6)	−0.0136 (6)	−0.0034 (5)
O3	0.0830 (8)	0.0635 (7)	0.0500 (6)	0.0195 (6)	−0.0134 (6)	−0.0004 (5)
O2	0.1054 (11)	0.0520 (7)	0.0711 (8)	0.0231 (7)	−0.0184 (7)	−0.0113 (6)
O5	0.0801 (8)	0.0648 (7)	0.0565 (7)	0.0263 (6)	−0.0129 (6)	−0.0064 (6)
O4	0.0916 (10)	0.0900 (9)	0.0540 (7)	0.0273 (8)	−0.0178 (7)	−0.0204 (7)
C1	0.0526 (9)	0.0504 (8)	0.0467 (8)	0.0198 (7)	−0.0020 (7)	0.0033 (6)
C9	0.0602 (9)	0.0507 (8)	0.0364 (7)	0.0064 (7)	0.0052 (6)	−0.0009 (6)
C20	0.0793 (12)	0.0528 (9)	0.0469 (8)	0.0146 (8)	−0.0085 (8)	−0.0076 (7)
C2	0.0545 (9)	0.0623 (10)	0.0634 (10)	0.0058 (8)	0.0093 (8)	−0.0027 (8)
C4	0.0731 (12)	0.0608 (10)	0.0593 (10)	0.0150 (9)	−0.0165 (9)	−0.0048 (8)
C21	0.0764 (11)	0.0501 (9)	0.0485 (9)	0.0166 (8)	−0.0030 (8)	−0.0031 (7)
C8	0.0636 (10)	0.0527 (9)	0.0391 (7)	0.0118 (7)	0.0060 (7)	−0.0027 (6)
C16	0.0625 (10)	0.0521 (9)	0.0444 (8)	0.0076 (7)	0.0016 (7)	0.0012 (7)
C6	0.0596 (11)	0.0764 (12)	0.0638 (11)	0.0145 (9)	0.0134 (9)	0.0064 (9)
C14	0.0741 (11)	0.0469 (8)	0.0538 (9)	0.0071 (8)	−0.0030 (8)	−0.0051 (7)
C3	0.0790 (13)	0.0689 (11)	0.0525 (9)	0.0179 (10)	0.0101 (9)	−0.0026 (8)
C7	0.0696 (11)	0.0597 (10)	0.0530 (9)	0.0255 (8)	−0.0050 (8)	−0.0020 (7)
C13	0.0677 (11)	0.0580 (10)	0.0519 (9)	0.0069 (8)	−0.0092 (8)	−0.0089 (7)
C10	0.0654 (10)	0.0520 (9)	0.0523 (9)	−0.0001 (8)	−0.0085 (8)	0.0035 (7)
C17	0.0805 (12)	0.0595 (10)	0.0437 (8)	0.0146 (9)	0.0008 (8)	−0.0081 (7)
C12	0.0654 (10)	0.0596 (9)	0.0394 (8)	0.0112 (8)	−0.0008 (7)	0.0027 (7)
C11	0.0722 (11)	0.0503 (9)	0.0649 (11)	0.0009 (8)	−0.0074 (9)	0.0092 (8)
C23	0.0639 (11)	0.0638 (10)	0.0619 (10)	0.0167 (9)	−0.0007 (8)	0.0004 (8)
C19	0.0631 (10)	0.0533 (9)	0.0476 (8)	0.0096 (8)	−0.0013 (7)	0.0019 (7)
C25	0.0647 (11)	0.0639 (11)	0.0734 (12)	0.0187 (9)	0.0002 (9)	0.0018 (9)
C18	0.0756 (11)	0.0576 (9)	0.0521 (9)	0.0214 (9)	0.0031 (8)	−0.0045 (7)
C22	0.0651 (11)	0.0633 (10)	0.0612 (10)	0.0189 (9)	0.0018 (8)	0.0005 (8)
C15	0.0650 (10)	0.0636 (10)	0.0461 (9)	0.0111 (8)	0.0001 (8)	0.0007 (7)
C5	0.0547 (10)	0.0754 (13)	0.0907 (15)	−0.0011 (9)	−0.0021 (10)	0.0000 (11)
C24	0.0730 (12)	0.0699 (11)	0.0655 (11)	0.0236 (10)	0.0027 (9)	−0.0020 (9)
C26	0.1012 (16)	0.0715 (13)	0.0904 (15)	0.0324 (12)	0.0009 (13)	0.0043 (11)
C27	0.1066 (19)	0.1006 (18)	0.126 (2)	0.0428 (15)	0.0030 (16)	0.0336 (16)

### Benzyl 4-{[4-(hexyloxy)benzoyl]oxy}benzoate Geometric parameters (Å, º)

**Table:**

O1—C8	1.3377 (19)	C7—H7A	0.9700
O1—C7	1.4603 (19)	C7—H7B	0.9700
O3—C15	1.372 (2)	C13—C12	1.381 (2)
O3—C12	1.398 (2)	C13—H13	0.9300
O2—C8	1.2022 (19)	C10—C11	1.377 (2)
O5—C19	1.355 (2)	C10—H10	0.9300
O5—C22	1.434 (2)	C17—C18	1.378 (2)
O4—O4	0.0000 (1)	C17—H17	0.9300
O4—C15	1.195 (2)	C12—C11	1.386 (2)
C1—C2	1.376 (2)	C11—H11	0.9300
C1—C6	1.377 (2)	C23—C22	1.496 (2)
C1—C7	1.499 (2)	C23—C24	1.520 (2)
C9—C10	1.387 (2)	C23—H23A	0.9700
C9—C14	1.388 (2)	C23—H23B	0.9700
C9—C8	1.489 (2)	C19—C18	1.389 (2)
C20—C21	1.372 (2)	C25—C24	1.503 (3)
C20—C19	1.391 (2)	C25—C26	1.508 (3)
C20—H20	0.9300	C25—H25A	0.9700
C2—C3	1.380 (2)	C25—H25B	0.9700
C2—H2	0.9300	C18—H18	0.9300
C4—C3	1.357 (3)	C22—H22A	0.9700
C4—C5	1.359 (3)	C22—H22B	0.9700
C4—H4	0.9300	C5—H5	0.9300
C21—C16	1.396 (2)	C24—H24A	0.9700
C21—H21	0.9300	C24—H24B	0.9700
C16—C17	1.390 (2)	C26—C27	1.486 (3)
C16—C15	1.473 (2)	C26—H26A	0.9700
C6—C5	1.381 (3)	C26—H26B	0.9700
C6—H6	0.9300	C27—H27A	0.9600
C14—C13	1.380 (2)	C27—H27B	0.9600
C14—H14	0.9300	C27—H27C	0.9600
C3—H3	0.9300		
			
C8—O1—C7	116.02 (12)	C10—C11—C12	118.91 (16)
C15—O3—C12	122.50 (13)	C10—C11—H11	120.5
C19—O5—C22	118.92 (13)	C12—C11—H11	120.5
C2—C1—C6	117.47 (15)	C22—C23—C24	111.89 (15)
C2—C1—C7	120.48 (16)	C22—C23—H23A	109.2
C6—C1—C7	122.03 (16)	C24—C23—H23A	109.2
C10—C9—C14	119.11 (15)	C22—C23—H23B	109.2
C10—C9—C8	121.85 (14)	C24—C23—H23B	109.2
C14—C9—C8	119.04 (14)	H23A—C23—H23B	107.9
C21—C20—C19	120.91 (15)	O5—C19—C18	124.48 (15)
C21—C20—H20	119.5	O5—C19—C20	115.99 (14)
C19—C20—H20	119.5	C18—C19—C20	119.53 (15)
C1—C2—C3	121.44 (17)	C24—C25—C26	113.46 (17)
C1—C2—H2	119.3	C24—C25—H25A	108.9
C3—C2—H2	119.3	C26—C25—H25A	108.9
C3—C4—C5	119.39 (17)	C24—C25—H25B	108.9
C3—C4—H4	120.3	C26—C25—H25B	108.9
C5—C4—H4	120.3	H25A—C25—H25B	107.7
C20—C21—C16	120.07 (15)	C17—C18—C19	119.30 (16)
C20—C21—H21	120.0	C17—C18—H18	120.4
C16—C21—H21	120.0	C19—C18—H18	120.4
O2—C8—O1	123.68 (15)	O5—C22—C23	108.17 (14)
O2—C8—C9	124.53 (15)	O5—C22—H22A	110.1
O1—C8—C9	111.79 (13)	C23—C22—H22A	110.1
C17—C16—C21	118.60 (15)	O5—C22—H22B	110.1
C17—C16—C15	118.11 (14)	C23—C22—H22B	110.1
C21—C16—C15	123.29 (15)	H22A—C22—H22B	108.4
C1—C6—C5	120.76 (18)	O4—C15—O3	123.77 (16)
C1—C6—H6	119.6	O4—C15—O3	123.77 (16)
C5—C6—H6	119.6	O4—C15—C16	125.12 (17)
C13—C14—C9	120.27 (15)	O4—C15—C16	125.12 (17)
C13—C14—H14	119.9	O3—C15—C16	111.09 (14)
C9—C14—H14	119.9	C4—C5—C6	120.76 (18)
C4—C3—C2	120.17 (18)	C4—C5—H5	119.6
C4—C3—H3	119.9	C6—C5—H5	119.6
C2—C3—H3	119.9	C25—C24—C23	115.27 (16)
O1—C7—C1	107.28 (12)	C25—C24—H24A	108.5
O1—C7—H7A	110.3	C23—C24—H24A	108.5
C1—C7—H7A	110.3	C25—C24—H24B	108.5
O1—C7—H7B	110.3	C23—C24—H24B	108.5
C1—C7—H7B	110.3	H24A—C24—H24B	107.5
H7A—C7—H7B	108.5	C27—C26—C25	115.4 (2)
C14—C13—C12	119.74 (16)	C27—C26—H26A	108.4
C14—C13—H13	120.1	C25—C26—H26A	108.4
C12—C13—H13	120.1	C27—C26—H26B	108.4
C11—C10—C9	121.17 (16)	C25—C26—H26B	108.4
C11—C10—H10	119.4	H26A—C26—H26B	107.5
C9—C10—H10	119.4	C26—C27—H27A	109.5
C18—C17—C16	121.59 (15)	C26—C27—H27B	109.5
C18—C17—H17	119.2	H27A—C27—H27B	109.5
C16—C17—H17	119.2	C26—C27—H27C	109.5
C13—C12—C11	120.79 (16)	H27A—C27—H27C	109.5
C13—C12—O3	114.70 (14)	H27B—C27—H27C	109.5
C11—C12—O3	124.38 (15)		
			
C6—C1—C2—C3	1.1 (3)	C9—C10—C11—C12	0.7 (3)
C7—C1—C2—C3	179.76 (16)	C13—C12—C11—C10	−0.3 (3)
C19—C20—C21—C16	−0.3 (3)	O3—C12—C11—C10	−175.85 (16)
C7—O1—C8—O2	1.7 (2)	C22—O5—C19—C18	2.2 (3)
C7—O1—C8—C9	−178.90 (13)	C22—O5—C19—C20	−177.34 (16)
C10—C9—C8—O2	−168.55 (17)	C21—C20—C19—O5	179.47 (16)
C14—C9—C8—O2	12.0 (2)	C21—C20—C19—C18	−0.1 (3)
C10—C9—C8—O1	12.1 (2)	C16—C17—C18—C19	−0.7 (3)
C14—C9—C8—O1	−167.38 (14)	O5—C19—C18—C17	−178.92 (17)
C20—C21—C16—C17	0.2 (3)	C20—C19—C18—C17	0.6 (3)
C20—C21—C16—C15	179.18 (17)	C19—O5—C22—C23	177.28 (15)
C2—C1—C6—C5	−0.7 (3)	C24—C23—C22—O5	−175.30 (16)
C7—C1—C6—C5	−179.28 (17)	O4—O4—C15—O3	0.0 (4)
C10—C9—C14—C13	−0.5 (3)	O4—O4—C15—C16	0.0 (5)
C8—C9—C14—C13	178.95 (15)	C12—O3—C15—O4	0.2 (3)
C5—C4—C3—C2	−0.6 (3)	C12—O3—C15—O4	0.2 (3)
C1—C2—C3—C4	−0.5 (3)	C12—O3—C15—C16	−178.07 (14)
C8—O1—C7—C1	−164.86 (14)	C17—C16—C15—O4	−6.6 (3)
C2—C1—C7—O1	81.28 (19)	C21—C16—C15—O4	174.47 (19)
C6—C1—C7—O1	−100.15 (18)	C17—C16—C15—O4	−6.6 (3)
C9—C14—C13—C12	0.9 (3)	C21—C16—C15—O4	174.47 (19)
C14—C9—C10—C11	−0.3 (3)	C17—C16—C15—O3	171.62 (15)
C8—C9—C10—C11	−179.74 (16)	C21—C16—C15—O3	−7.3 (2)
C21—C16—C17—C18	0.3 (3)	C3—C4—C5—C6	1.1 (3)
C15—C16—C17—C18	−178.71 (17)	C1—C6—C5—C4	−0.4 (3)
C14—C13—C12—C11	−0.5 (3)	C26—C25—C24—C23	−174.58 (18)
C14—C13—C12—O3	175.46 (15)	C22—C23—C24—C25	179.98 (17)
C15—O3—C12—C13	148.39 (16)	C24—C25—C26—C27	−176.2 (2)
C15—O3—C12—C11	−35.8 (3)		

### Benzyl 4-{[4-(hexyloxy)benzoyl]oxy}benzoate Hydrogen-bond geometry (Å, º)

*Cg*1 and *Cg*3 represent the centroids of the C1–C6 and C16–C21 aromatic rings, respectively.

**Table:**

*D*—H···*A*	*D*—H	H···*A*	*D*···*A*	*D*—H···*A*
C10—H10···O1	0.93	2.41	2.730 (2)	100
C11—H11···O4	0.93	2.40	2.851 (2)	109
C7—H7*B*···*Cg*1^i^	0.97	2.92	3.602 (2)	128
C22—H22*B*···*Cg*3^ii^	0.97	2.99	3.810 (2)	143
C27—H27*A*···*Cg*1^iii^	0.96	2.98	3.791 (3)	143

Symmetry codes: (i) −*x*+4, −*y*, −*z*+2; (ii) *x*−1, *y*, *z*; (iii) −*x*+1, −*y*+1, −*z*+1.
